# The Hypothesis that the Genetic Code Originated in Coupled Synthesis of Proteins and the Evolutionary Predecessors of Nucleic Acids in Primitive Cells

**DOI:** 10.3390/life5010467

**Published:** 2015-02-11

**Authors:** Brian R. Francis

**Affiliations:** Department of Molecular Biology, University of Wyoming, Laramie, WY 82071, USA; E-Mail: brianrf@uwyo.edu; Tel.: +1-307-460-0408

**Keywords:** origin genetic code, primitive cell, coupled protein nucleic acid synthesis

## Abstract

Although analysis of the genetic code has allowed explanations for its evolution to be proposed, little evidence exists in biochemistry and molecular biology to offer an explanation for the origin of the genetic code. In particular, two features of biology make the origin of the genetic code difficult to understand. First, nucleic acids are highly complicated polymers requiring numerous enzymes for biosynthesis. Secondly, proteins have a simple backbone with a set of 20 different amino acid side chains synthesized by a highly complicated ribosomal process in which mRNA sequences are read in triplets. Apparently, both nucleic acid and protein syntheses have extensive evolutionary histories. Supporting these processes is a complex metabolism and at the hub of metabolism are the carboxylic acid cycles. This paper advances the hypothesis that the earliest predecessor of the nucleic acids was a β-linked polyester made from malic acid, a highly conserved metabolite in the carboxylic acid cycles. In the β-linked polyester, the side chains are carboxylic acid groups capable of forming interstrand double hydrogen bonds. Evolution of the nucleic acids involved changes to the backbone and side chain of poly(β-d-malic acid). Conversion of the side chain carboxylic acid into a carboxamide or a longer side chain bearing a carboxamide group, allowed information polymers to form amide pairs between polyester chains. Aminoacylation of the hydroxyl groups of malic acid and its derivatives with simple amino acids such as glycine and alanine allowed coupling of polyester synthesis and protein synthesis. Use of polypeptides containing glycine and l-alanine for activation of two different monomers with either glycine or l-alanine allowed simple coded autocatalytic synthesis of polyesters and polypeptides and established the first genetic code. A primitive cell capable of supporting electron transport, thioester synthesis, reduction reactions, and synthesis of polyesters and polypeptides is proposed. The cell consists of an iron-sulfide particle enclosed by tholin, a heterogeneous organic material that is produced by Miller-Urey type experiments that simulate conditions on the early Earth. As the synthesis of nucleic acids evolved from β-linked polyesters, the singlet coding system for replication evolved into a four nucleotide/four amino acid process (AMP = aspartic acid, GMP = glycine, UMP = valine, CMP = alanine) and then into the triplet ribosomal process that permitted multiple copies of protein to be synthesized independent of replication. This hypothesis reconciles the “genetics first” and “metabolism first” approaches to the origin of life and explains why there are four bases in the genetic alphabet.

## 1. Introduction

Despite considerable research devoted to studying how life started on Earth, no widely accepted theory of the origin of life has emerged. Such a theory would have to explain how an initial simple system was established and how the simple system evolved into the complex biological system. At the present time, there are many hypotheses that have varying degrees of support for different aspects of the problem. The most widely accepted hypothesis among biologists, the RNA world hypothesis, still has strong supporters [1] but difficulties with the hypothesis are recognized, especially the problem of synthesizing RNA in the absence of enzymes [2,3,4,5,6,7]. The RNA world hypothesis is central to the “genetics first” approach to the origin of life. An alternative approach involves a system in which cellular components coevolved [5,7,8]. Because nucleic acids are complex polymers and ribosomal protein synthesis is a highly complex process, a particularly challenging question is how did proteins and nucleic acids coevolve?

Many years ago, Lipmann [9] wrote, “In the domain of the transfer of genetic information I felt naïvely that, before translation into amino acid sequences could become the target of the genetic code, the extraordinary functional pliability of proteins would have to be established”. Given what is now known about ribosomal protein synthesis, it does not seem to be such a naïve observation. Previously, I proposed that ribosomal protein synthesis evolved from coupled synthesis of nucleic acids and proteins. This proposal allowed coded protein synthesis to precede ribosomal protein synthesis [8]. Subsequently, I extended this hypothesis to describe how the coupled process evolved with continuity into ribosomal protein synthesis and, in so doing, provided an alternative to the RNA world hypothesis [5]. In order to develop a coevolutionary theory, the need for coded protein synthesis prior to ribosomal protein synthesis has assumed greater importance. In this paper, I present the argument for coupled protein and nucleic acid synthesis in an updated and different way from that which was published in 2000.

## 2. Autocatalysis in the Origin of Life

What seems to be a point of agreement in a contentious field is that the origin of life involved some type of simple autocatalytic system in which the products of reactions catalyzed their own synthesis. Proteins often acting together with coenzymes/cofactors perform almost all of the biological catalysis. The complex autocatalytic system of biology involves symmetry breaking in which amino acids of only one configuration, l-amino acids, are incorporated into proteins to provide reproducible structures that catalyze reactions in the cell including synthesis of the l-amino acids themselves as well as numerous other chiral molecules including d-ribose in RNA and d-deoxyribose in DNA. All chirality in biochemistry begins with proteins. The enzymes involved are the aminoacyl-tRNA synthetases that preferentially bind l-amino acids. A reasonable assumption is that the simple autocatalytic system for the origin of life used proteins in symmetry breaking and catalysis. I will return to this issue later.

## 3. Energetics, Genetics, Catalysis and Membranes

Four major activities of cells are energy transduction, replication of genetic polymers, catalysis of chemical reactions, and formation of the membranes that define the three dimensional cellular space and the interaction of the cell with the environment. Each of these activities has to be addressed in a theory of the origin of life. It may be that replication of genetic polymers was not an initial requirement—perhaps growth without replication was possible at the beginning—but soon after the initiation of the simple system, genetic polymers would need to evolve so that selection could occur. How could a simple system incorporate these four activities? Using chemistry and biochemistry as a guide, I will suggest what these activities might have been leading to a model for a primitive cell.

## 4. Thioesters Were Used for Synthesis in Primitive Cells

Maps of cellular metabolic pathways show a central hub, the carboxylic acid cycles, a major spoke, gluconeogenesis/glycolysis, and a large number of other pathways leading from the hub and spoke to synthesis and degradation of nucleic acids, amino acids, lipids, carbohydrates, coenzymes and cofactors. Proponents of the “metabolism first” hypothesis for the origin of life suggest that reactions such as those found in the reverse citric acid cycle, the acetogenic pathway, or the methanogenic pathway may have occurred prior to the evolution of genetic polymers and enzymes [10,11,12,13,14,15,16,17]. The carboxylic acid cycles include molecules that form thioester bonds between carboxylic acids and coenzyme A (CoA), such as acetyl-CoA, succinyl-CoA and malyl-CoA. After the discovery of CoA and the role of phosphopantetheine in fatty acid and non-ribosomal peptide synthesis. Lipmann [9] proposed that use of thioester bonds provided a simpler biosynthetic mechanism for peptide bond synthesis than ribosomal synthesis. Subsequently, the idea of an early metabolic system powered by thioesters has been championed by other researchers [18,19,20,21]. CoA has an unusual and complex structure. Simple thiols such as CH_3_SH formed from the reaction of CO_2_, H_2_S, and FeS [22] may have been used initially for thioester synthesis. The alternative view is that phosphate is so extensively used in metabolism that it had to be involved from the beginning [23,24,25]. If phosphate was not initially required, the model for a primitive cellular system is greatly simplified because there is no need for phosphate uptake and utilization.

## 5. Thioester Bonds Were Used to Form Ester and Amide Bonds

Given the choice, acyl groups move from thiol to hydroxyl or amino groups [26]. Therefore, generation of thioester bonds could have driven formation of ester and amide bonds. For example, aminoacyl-thioesters form oligopeptides in the absence of enzymes [27,28], and α-thioglutamic acid oligomerizes [29,30]. A glyceroyl thioester oligomerizes chemically to form the polyester, oligoglyceric acid [31], and in biology ester bonds in poly(β-l-hydroxybutyric acid) (PHBA) are formed from β-l-hydroxybutyryl-CoA [32]. Therefore, an energy flux in primitive cells producing thioester bonds could have led to formation of ester and amide linked polymers in a simple system.

## 6. Energy Transduction in Biology

What was the earliest mechanism by which cells were energized? The following analysis of existing mechanisms suggests it was a redox gradient across a membrane. Energy transduction by respiration is an ancient process [33]. In “modern” cells, it is extraordinarily complicated, requiring formation of ion gradients across cell membranes and use of these gradients to drive ATP synthesis. The ion gradients are generated using the energy derived from redox processes in which electrons from oxidation of molecules such as NADH are transported to a receptor such as oxygen. Specialized lipid molecules, including ubiquinone and methanophenazine, and complexes of membrane proteins and cofactors, particularly heme groups and FeS clusters, are involved in electron transport and pumping of H^+^ or Na^+^ across a membrane to establish the gradient. A variety of electron donors and receptors are used in biochemistry. Conversion of the energy in a H^+^ or Na^+^ gradient into ATP synthesis uses a complex of proteins acting as a molecular motor, ATP synthase. The ion gradient appears to be secondary to the redox gradient suggesting that the redox gradient is the ancient means by which cells were energized. An alternative view suggested by geochemistry is that an acidic carbon dioxide-rich cool ocean and an alkaline hydrogen-rich warm interior of a metal sulfide deposit on the ocean floor near a hydrothermal system provided naturally occurring gradients across inorganic membranes [17,34].

How was a redox gradient established in a simple cell? An important feature of respiration is that the sites of the oxidation and reduction reactions are separated. In the case of NADH as electron donor and oxygen as electron acceptor, electrons can enter and leave the electron transport system on the inside of the membrane. The slow rate of reaction of NADH with oxygen allows this to occur. Generally, electrons can enter or leave electron transport systems from the inside or the outside of a membrane. In principle, a simple redox system would involve separation of the oxidation and reduction events by a membrane with transfer of electrons across the membrane into a primitive cell. The inside of the cell would therefore act as a cathode. Reduced products could accumulate inside primitive cells. What were the oxidation and reduction reactions involved in energy transduction in the simplest cells? The early Earth was anaerobic. Some of the possible electron donors on the primitive earth suggested by biochemistry are hydrogen, hydrogen sulfide, Fe(II), and sulfite. The Fe(II) is often part of an FeS cluster. The electron donor used previously [8] was sulfite produced by dissolving volcanic sulfur dioxide in water. I will use it again in this study.

H_2_S → S^0^ + 2H^+^ + 2e^−^

H_2_ → 2H^+^ + 2e^−^

SO_3_^2−^ + H_2_O → SO_4_^2−^ + 2H^+^ + 2e^−^

Fe(II) → Fe(III) + e^−^

[(Fe_4_S_4_)(SR)_4_]^3−^ → [(Fe_4_S_4_)(SR)_4_]^2−^ + e^−^

What was the earliest electron acceptor? Some of the possibilities are shown below.

Fe(III) + e^−^ → Fe(II)

[(Fe_4_S_4_)(SR)_4_]^2−^ + e^−^ → [(Fe_4_S_4_)(SR)_4_]^3−^

RS-SR + 2H^+^ + 2e^−^ → 2RSH

S^0^ + 2H^+^ + 2e^−^ → H_2_S

CO + 2H^+^ + 2e^−^ → H_2_CO

CO_2_ + 2H^+^ + 2e^−^ → HCOOH

NO_2_^−^ + 8H^+^ + 6e^−^ → NH_4_^+^ + 2H_2_O

Two of the simplest electron acceptors in biochemistry are a disulfide bond and Fe(III) with Fe(III) often part of an FeS cluster, such as [(Fe_4_S_4_)(SR)_4_]^2−^ (R = organic group). Sulfur-based respiration is commonly found in prokaryotes, especially hyperthermophilic archaea [35]. Three other possible electron receptors are CO, CO_2_, and NO/NO_2_^−^. The atmosphere of the early Earth may have contained CO as well as CO_2_ [36], allowing the possibility that CO was reduced to organic compounds inside cells. CO with FeS and thiols at elevated temperatures and pressures produces organic molecules including pyruvic acid via intermediary formation of Fe-carbonyl compounds [37]. FeS clusters, CO, and H_2_ in the gas phase form formaldehyde and methanol [38]. Biochemically, reducing (“fixing”) CO_2_ requires complex enzyme catalyzed reactions [39]. CO_2_ is a very stable molecule, and reduction to formate by H_2_ is thermodynamically unfavorable, although the reaction of H_2_ with bicarbonate is favorable [40]. In the ocean hydrothermal vent scenario, it is thought that the reactions of the acetogenic or methanogenic pathways for CO_2_ fixation mimic ancient non-enzymatically catalyzed reactions [16,34]. H_2_S can reduce CO_2_ when FeS is oxidized to FeS_2_ [10]. If the inside of a primitive cell acted as a cathode, CO_2_ fixation could have been accomplished similar to the electrolytic process known as electrocarboxylation [40], as in electrolytic reduction of CO_2_ to formic acid catalyzed by [Fe_4_S_4_(SR)_4_]^2−^ [41]. Simple redox reactions that occur chemically include oxidation of sulfite to sulphate and thiols to disulfides by Fe(III) [42,43]. In this proposal I will use FeS as the primary electron acceptor. NAD(P)^+^/NAD(P)H and FAD/FADH_2_ are commonly used redox reagents in biochemistry but their complex syntheses make it unlikely that they were present in an early redox system.

Previously, I assumed that early cells obtained reduced nitrogen for amino and amide chemistry by diffusion across primitive membranes of cyanides, and the thioamides and amides resulting from the reaction of cyanides with H_2_S and water [8]. Diffusion of molecules such as formamide and acetamide remains an attractive possibility. An alternative explanation is that ammonium ions were produced by reduction of NO or nitrite inside cells. NO has been proposed as the first deep electron sink for respiration [44]. NO produced in the atmosphere could have undergone reactions leading to accumulation of nitrite and nitrate in water [45]. If primitive cells were leaky to ions, nitrite and nitrate could diffuse across membranes and be reduced to ammonia using Fe(II) or FeS [46,47,48]. Ethanethiol and other simple thiols react with NO or nitrite to produce S-nitroso compounds [49]. Glutathione SH groups undergo the same reaction with NO in biochemistry [50]. If primitive cells were restricted towards ion transport, nitrite could have diffused into cells as the uncharged S-nitroso derivatives of thiols. Nitrite, released from the S-nitroso compound by hydrolysis inside the cells with formation of a metal thiolate, could be reduced to ammonia.

## 7. Formation of the Earliest Cell Membranes

If primitive cells were energized by electron transport across a cell membrane, what material constituted the membrane? Two very different views of the origin of cell membranes have been proposed. Both approaches seek to prevent dilution of metabolites in water by compartmentalization. One view is that the original cell membranes were formed from organic materials. Synthesis of membrane phospholipids for biological membranes is a complex biochemical process whether it involves the linkage of 2-carbon units from acetyl-CoA that occurs in bacteria and eukarya or the linkage of 5-carbon isoprene units that occurs in archaea. Lipids of this type were probably not available on the primitive Earth. Organic molecules for cell membranes could have been delivered to the early Earth by asteroids, meteors, comets, and interstellar dust particles (IDPs). Among a diverse range of organic molecules in meteorites are single chain amphiphiles such as fatty acids that are considered to be good candidates for prebiotic vesicles because they have higher permeability to ions than the double chain amphiphiles found in modern cell membranes [51,52,53,54,55]. Polyaromatic hydrocarbons (PAH) that are abundant in meteorites and IDPs [56] may have been included in vesicle membranes. Organic molecules could have formed on the early Earth from the interaction of energy sources such as UV light and lightning with volcanic gases, as demonstrated by Miller-Urey type experiments ([57], references therein), and accumulated in ponds near volcanoes. The other view is that the original membranes were formed from semi-porous inorganic materials, particularly metal sulfides, in deposits at hydrothermal vents on the ocean floor. Lipid-like molecules are not exuded from modern day vents. Lipid membrane synthesis is proposed to have occurred within metal sulfide bubbles eventually enabling cells to exist free of the sulfide deposits [17]. Another proposal is that the semi-porous metal sulfide deposits were at the Earth’s surface associated with volcanic fields [58,59].

Membrane biogenesis involves insertion of newly synthesized membrane components, primarily lipids and proteins, into existing membranes. Accordingly, the easiest mechanism to consider for membrane biogenesis in primitive cells involves insertion of newly synthesized membrane components into pre-existing organic membranes. This seems more reasonable than creation of a *de novo* organic membrane within an inorganic membrane. Although most attention has been afforded to primitive lipid vesicles based on biological lipids, another possibility is that the organic material was formed from a different type of organic material. The most recognized products of Miller-Urey type experiments are carboxylic acids including amino and hydroxy acids [60], but there is also a water insoluble heterogeneous mixture of organic molecules called tholin [61]. Tholin is also produced by UV irradiation of gas mixtures and is found on the surfaces of Jupiter’s moons, Ganymede and Callipso [62], and in the haze of Saturn’s moon, Titan [63,64,65]. Tholin can form laminated structures that may have formed the earliest cells [66]. Extractable components of tholin have a C_i_H_j_N_k_ core terminally linked to amino and carboxylic acids [67]. Miller-Urey type experiments have been performed with H_2_S in the mixture [60,68,69,70]. Tholin obtained from these experiments contains thiol groups [71]. Observations of the tholin in the haze around Titan show a complex mixture of hydrocarbons, including PAH, nitriles [64], and polyynes [65]. Polymers made from hydrogen cyanide, a major intermediate of amino acid synthesis in Miller-Urey type experiments, have an unsaturated structure in which the dominating polymers contain polyimine chain-like structures [72,73]. Therefore, tholin is a material with a high level of unsaturation including conjugated bonds and heterocyclic and polyaromatic ring components.

## 8. Energization of Primitive Cells Using Tholin as an Electron Transporting Organic Membrane

Accepting that the earliest electron transport system involved transport from the outside to the inside of a membrane, how could electrons have been transported across an organic membrane in the absence of complicated proteins/cofactors and specialized lipids that are found in biology (reviewed in [24,74])? One possibility comes from the observation that PAHs form quinone type molecules upon photoirradiation in the presence of water. These types of molecules might have accumulated in organic material on the early Earth and functioned in membranes like ubiquinone [75]. Another idea is that electron transport could have used a relatively simple type of cofactor such as an FeS cluster. Electron transport by FeS clusters bound to proteins generally occurs in protein domains associated with but not in membranes. Electrons can tunnel between FeS clusters [76]. It is possible that electrons were transported by FeS clusters embedded in a thiol bearing organic material. A third mechanism might involve the unsaturated components of tholin material. “Electrical” organic polymers contain conjugated unsaturated bonds [77]. Main chain peptide bonds can transport electrons [78]. Aromatic side chains in proteins can facilitate electron transport within and between proteins. An example is found in ribonucleotide reductase class I where the side chains of Tyr and Trp (using three letter abbreviations for amino acids) facilitate electron transfer from Tyr to Cys [79]. The nature of tholin suggests that it was an organic material that could transport electrons. The evolutionary pathway for membrane biogenesis would involve insertion into the tholin-containing membranes of specialized proteins, cofactors and lipids for electron transport, together with proteins performing other functions and lipids that did not transport electrons, eventually eliminating the need for tholin.

## 9. Iron Sulfide in Origin of Life Scenarios

If tholin formed primitive electron-transporting cell membranes, what was the electron acceptor and how was the energy from electron transport harnessed for synthesis within the cells? Because thioesters played a central role in primitive cell synthesis, the question becomes: How was electron transport linked to thioester synthesis? Before enzymes were available, this would probably have required an alternate type of catalyst. Building on an earlier proposal by Koch and Schmidt [80], I proposed that the first cells were made when tholin enclosed FeS using its thiol groups for binding to FeS. Within these cells, FeS particles or FeS clusters provided sites for catalysis of chemical reactions including sites for thioester synthesis [8]. Next, I will mention alternative ideas regarding metal sulfides and thioester synthesis in the origin of life, and in the following two sections, provide background on the chemistry and biochemistry of FeS clusters and thioesters that will lead to the proposal of a simple FeS-catalyzed mechanism for thioester synthesis.

Many studies of the origin of life have focused on mineral surfaces as binding and catalytic sites for small molecules [14]. Because cofactors made from metal sulfides are often the sites of redox and catalytic activities in proteins and FeS, NiS, and other sulfides are likely to have been present on the primitive Earth in association with volcanic or hydrothermal activity, reactions involving transition metal sulfides, particularly FeS, have been extensively studied in simulations of reactions on the primitive Earth. Wächtershäuser [21,81] proposes a chemoautotrophic origin of life in an iron-sulfur world. The favored site for the origin of life is a hydrothermal vent where metal sulfides are deposited. Experimental reactions under hydrothermal conditions using H_2_S as electron source, CO as carbon source, and NiS/FeS as catalyst produce a thioester, CH_3_COSCH_3_ [82]. In these studies, FeS is not a catalyst because it is converted to FeS_2_. In a related proposal, FeS forms primitive cell membranes allowing concentration of reaction products in hydrothermal deposits [17]. In these cells, it is proposed that reactions analogous to the biological Wood-Ljungdahl acetogenic pathway for thioester synthesis could have occurred in the absence of enzymes using H_2_ and CO_2_ energized by redox, proton, and temperature gradients [34]. Enzymatically, this is a complicated pathway. Cofactors involved are folic acid, a corrin ring, and an FeNiS complex in a process that involves reduction of CO_2_ separately to CO and a methyl group, which then react with CoA to form acetyl-CoA [83].

## 10. FeS Cluster Biochemistry and Chemistry

FeS clusters bound to proteins, exemplified by [Fe_4_S_4_]^2+^, have both redox and non-redox functions [84]. The ability of FeS clusters to carry different charges allows FeS proteins to be involved in redox reactions, often in electron transport chains. An extensively studied class of FeS proteins is ferredoxin, a mobile carrier of electrons, which has Fe_4_S_4_ or Fe_2_S_2_ clusters bound to cysteine side chains as thiolate ligands. Ferredoxin is thought to be an ancient FeS protein in which the clusters cycle between reduced and oxidized forms through one-electron transfers [85,86]. Use of ferredoxin as a reducing agent is demonstrated by pyruvate synthase (pyruvate ferredoxin oxidoreductase)-catalyzed reductive carboxylation of acetyl-CoA to form pyruvate in the presence of thiamine pyrophosphate (TPP) [87,88].

Iron in FeS proteins is usually coordinated by Cys thiolate anions but oxygen can provide ligands to Fe in FeS clusters during enzymatic catalysis [89]. In the active site of aconitase, one of the Fe in a Fe_4_S_4_ cluster binds to water, a carboxylate and a hydroxyl group during interconversion of citrate and isocitrate in a catalytic mechanism that does not involve a redox component. Fumerase uses FeS clusters to catalyze interconversion of malic and fumaric acids [90]. Aconitase and fumarase are examples of a wide range of dehydratases with FeS clusters that catalyze dehydration reactions producing carbon-carbon double bonds [89].

FeS clusters like [Fe_4_S_4_(SR)_4_]^n−^ and [Fe_2_S_2_(SR)_2_]^n−^ that are similar to those found in FeS proteins have been synthesized chemically [91,92,93]. In the absence of proteins, they tend to be unstable and insoluble in water [94], but are stabilized by the presence of thiols [95]. Their reactions have mostly been studied in organic solvents. As in biochemistry, Fe_4_S_4_(SR)_4_ can exist in different ionic states with more negatively charged clusters acting as reducing agents [92]. Reactions that do not involve electron transfer can produce clusters in which Fe is bound to oxygen. For example, the reaction of Fe_4_S_4_(SR)_4_ with acetic acid produces an acetate-bound cluster and RSH. Reaction with the more electrophilic carbonyl of acetyl chloride produces a thioester, CH_3_COSR [91].

## 11. Biochemical Reduction of Disulfides and Thioester Formation

The ability of the thiol group to carry an acyl group as a thioester and be oxidized to a disulfide provides the most obvious simple link in early cells between electron transport and chemical energy for biosynthesis. The dithiol/disulfide redox reaction is reversible under mild conditions. Two enzymes illustrate a connection between FeS cluster chemistry and disulfide reduction. Ferredoxin-thioredoxin reductase (FTR) catalyzes transfer of electrons from reduced ferredoxin to thioredoxin [96]. In the mechanism of the reaction, two electrons, one from a reduced ferredoxin and one from the FeS cluster in FTR, are used to cleave an adjacent cystine. The thiolate produced attacks the disulfide of thioredoxin. A second one-electron transfer from reduced ferredoxin completes the reduction of the thioredoxin disulfide. A key feature of the reaction is the ability of the Fe_4_S_4_ cluster of FTR to vary its charge and allow one Fe to adopt five-fold coordination during the reaction. Heterodisulfide reductase (HDR) catalyzes two one-electron transfers to the disulfide formed between coenzyme B (CoB) and coenzyme M (CoM). CoB-S-S-CoA is the terminal electron receptor in methanogenesis [97]. A mechanism similar to FTR is involved but the reaction is not mediated by a cystine. CoMS-SCoB interacts directly with a Fe_4_S_4_ cluster and the sulfur of CoM binds to the cluster [98,99].

The equilibrium for thioester synthesis from a thiol and carboxylic acid is highly unfavorable for formation of a thioester and water. Consequently, thioester synthesis requires coupling to an energy releasing reaction. Mechanisms for thioester biosynthesis have redox components. In synthesis of acetyl-CoA from pyruvate catalyzed by pyruvate dehydrogenase, a critical reaction is between a lipoamide molecule bearing a disulfide bond and an acetaldehyde formed by decarboxylation of pyruvate that is bound to TPP as a hydroxyethyl group. Formation of the acetyl thioester derivative of lipoamide involves oxidation of the acetaldehyde to an acetyl group and reduction of the lipoamide disulfide. Subsequently, the acetyl group is transferred to CoA. This reaction provides a biochemical link between disulfide reduction and thioester synthesis. Formation of a thioester from an aldehyde is also involved in the mechanism of the reaction catalyzed by glyceraldehyde-3-phosphate dehydrogenase. In most organisms, the thioester is produced by oxidation of a hemithioacetal formed between a Cys-SH and glyceraldehyde-3-phosphate using NAD^+^ as the oxidizing agent, but in some cases ferredoxin is used as the electron acceptor instead of NAD^+^ [100,101].

One can conclude that thioester synthesis in primitive cells was likely to be associated with FeS redox chemistry and a simple mechanism for thioester synthesis could have involved coupling to reduction of a disulfide bond.

## 12. A Proposal for Primitive Cellular Synthesis of Thioesters

From the characteristics of reactions involving thiols, disulfides, carboxylic acids, and FeS compounds, and the analysis of chemical and biochemical thioester syntheses, it is plausible that FeS chemistry played a role in prebiotic thioester synthesis. My proposal is that electrons generated from oxidation reactions outside primitive cells were transported and used inside the cells for formation of thioesters by the following reaction mediated by FeS clusters or the surface of an FeS particle. Intermediate formation of an aldehyde by reduction of the carboxylic acid prior to reaction with the disulfide may not have been necessary.

RS-SR + 2e^−^ + 2H^+^ + R’COOH → RSH + R’COSR + H_2_O

Using sulfite oxidation as the electron source, the overall reaction is

RS-SR + SO_3_^2−^ + R’COOH → RSH + R’COSR + SO_4_^2−^

**Figure 1:**
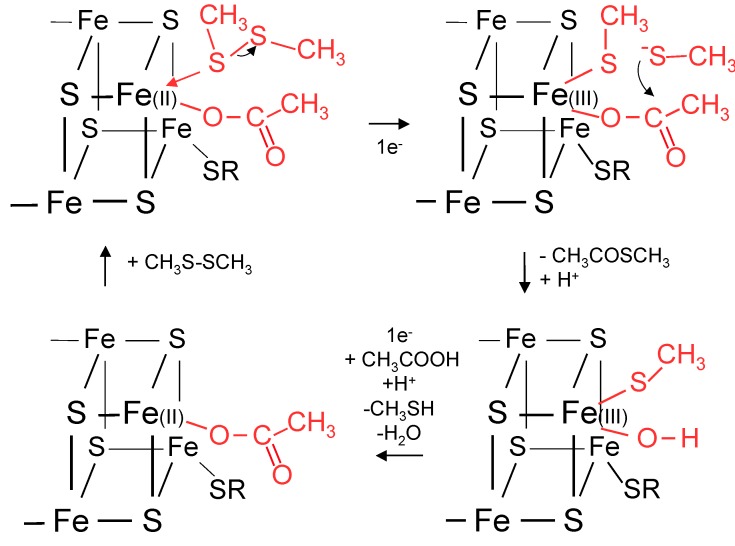
Mechanism proposed for catalysis of thioester synthesis in primitive cells by an FeS particle. A thiolate anion from reduction of a disulfide, CH_3_SSCH_3_, reacts with acetate bound to Fe of an FeS particle to form a thioester, a thiol, and hydroxylated Fe. Using the mechanisms of FTR and HDR as models, one of the two electrons for reaction with the disulfide is provided by electron transport from an oxidation reaction and the other electron by conversion of Fe(II) to Fe(III). Transfer of another electron regenerates the FeS binding site and loss of water permits a new round of thioester synthesis.

A proposal for acetyl-thioester synthesis based on the HDR/FTR mechanism using the surface of an FeS particle is given in Figure 1. In the presence of thiols and carboxylic acids, FeS could have provided a simple catalytic surface for thioester synthesis without formation of FeS_2_.

## 13. Diffusion of Small Polar and Nonpolar Molecules across Primitive Cell Membranes

Simple molecules such as water, carbon dioxide, oxygen, nitrogen, hydrogen, hydrogen sulfide, and nitric oxide diffuse across biological membranes but transport of ions and organic molecules involves integral membrane protein transporters or channels. If primitive cells were leaky to ions, as they would be if they were composed of fatty acids [52], and many different molecules and ions were present in the environment, the number of possible cellular components would have been high. Miller-Urey type experiments produce a large number of molecules including carboxylic acids, amino acids, and hydroxyacids although their relative amounts vary widely. It is thought that this was highly beneficial for the origin of life because these molecules could have been assembled into macromolecules by the action of condensing agents like carbonyl sulfide [102] or through wet dry cycles using salts [103] to produce a wide variety of possible catalytic activities. In this line of reasoning, cellular membrane evolution involved increasing the barrier provided to ion permeability [25,104]. On the other hand, leaky membranes would have meant that the inside and outside of a cell would have been equilibrated with regard to small molecules, which would have made it difficult to synthesize macromolecules. Those who advocate an inorganic membrane for early cells refer to its semiporous nature, which would allow diffusion of CO_2_ from the environment, and have the inorganic compartment accessible to molecules from within the metal sulfide deposits [17,58].

I favor early organic membranes that presented a barrier to the passage of ions including amino acids, but were permeable to simple non-polar and polar molecules including H_2_S, CH_3_SH, CO_2_, CO, HCONH_2_ and CH_3_SNO. It is difficult to stop protons from leaking across lipid bilayer membranes [105], and presumably the earliest organic membranes did not have the ability to prevent proton transfer. At first sight, it seems odd that amino acids might be available in the environment but not used inside cells, but cells that were not leaky to ions would not be equilibrated with the external environment, energy from redox reactions could be used to maintain cells away from thermodynamic equilibrium, and the buildup of macromolecules could proceed. Monomers, including amino acids, required for assembly into macromolecules would have been synthesized inside cells from diffusible materials.

## 14. Reduction and Carboxylation Reactions inside Primitive Cells

In addition to thioester synthesis, other reactions might have occurred in these primitive cells either at the FeS surface or by reduction reactions with thiols in solution that would be required to continue thioester synthesis by recycling of thiols to disulfides. Possible reactions, listed in Table 1, are described below.

Reductions of CO, CO_2_, and NO involving FeS have been mentioned previously. Another potential reduction reaction would be conversion of a carboxyl group to an aldehyde. Biochemistry often overcomes the energy barrier for this reaction by intermediate formation of an acyl phosphate before reduction with NAD(P)H, but fatty acid reduction to an aldehyde uses a thioester with CoA as substrate [106]. De Duve [107] speculates that thioester reduction to an aldehyde, perhaps using FeS as a catalyst, was the earliest mechanism. Direct evidence for aldehyde formation from thioesters catalyzed chemically by FeS or FeS clusters has not yet been reported. The reaction would require transfer of hydride for C–H bond formation. However, lactic acid synthesis from pyruvic acid catalyzed by FeS/H_2_/H_2_S produces a C–H bond [108]. The mechanism for this reaction indirectly involves H_2_. Reduction of α-keto acids is also found using FeS/H_2_S [109]. The Fukuyama reduction reaction that is used chemically for aldehyde synthesis from thioesters involves formation of an acyl-Pd-H intermediate [110]. An acyl-Fe-H intermediate may be involved in FeS catalyzed lactic acid synthesis, but a carboxylate-Fe-H intermediate is also possible.

**Table 1:** Possible reactions in a primitive cell formed by enclosure of an FeS particle in tholin.

Activity	Reaction
1. Thioester synthesis	CH_3_COOH + 2e^−^ + 2H^+^ + RSSR → CH_3_COSR + RSH + H_2_O
2. CO reduction	CO + 2e^−^ + 2H^+^ → H_2_CO
3. CO_2_ reduction	CO_2_ + 2H^+^ + 2e^−^ → HCOOH
4. Nitrite reduction	NO_2_^−^ + 8H^+^ + 6e^−^ → NH_4_^+^ + 2H_2_O
5. Reductive carboxylation	CH_3_COSR + 2e^−^ + CO_2_ + 2H^+^ → CH_3_COCOOH + RSH
6. Reductive amination	CH_3_COCOOH + 2RSH + NH_3_ → CH_3_CH(NH_2_)COOH + RSSR + H_2_O
	CH_3_COCOOH + 2e^−^ + 2H^+^ + NH_3_ → CH_3_CH(NH_2_)COOH + H_2_O
7. Glycine synthesis	H_2_CO + NH_3_ + CO_2_ + 2RSH → HOOCCH_2_NH_2_ + H_2_O + RSSR
8. Claisen condensation	CH_3_COSR + HCOCOOH → HOOCCH(OH)CH_2_COSR
9. Carboxylation	CH_3_COCOOH + CO_2_ → HOOCCH_2_COCOOH
10. Reduction of ketone	HOOCCOCH_2_COOH + 2e^−^ + 2H^+^ → HOOCCH(OH)CH_2_COOH
11. Reductive thiolation	HCOCOOH + 2e^−^ + H_2_S + 2H+ → HSCH_2_COOH + H_2_O
12. Aldol condensation	2CH_3_COCOOH → HOOCC(CH_3_)OHCH_2_COCOOH
13. Acyl group transfer	R’COSR + R”OH → R’COOR” + HSR
	R’COSR + R”NH_2_ → R’CONHR” + HSR
14. β-Polyester synthesis	–(COCH_2_CH(COOH)O)_n_– + RSCOCH_2_CH(COOH)OH → –(COCH_2_CH(COOH)O)_n+1_– + RSH
15. Polypeptide synthesis	–(NHCHR’CO)_n_– + H_2_NCHR’COSR → –(NHCHR’CO)_n+1_– + HSR

Early expansion of metabolism required the synthesis of three- and four-carbon molecules. Pyruvic acid, a pivotal three-carbon molecule in biochemistry, is made by carboxylation of acetaldehyde bound to TPP using reduced ferredoxin for two one-electron transfers catalyzed by pyruvate ferredoxin oxidoreductase [111,112]. A reductive carboxylation reaction of this type using dithionite as electron donor is chemically catalyzed by an FeS complex but the reaction is performed in a partially aqueous solution [113]. An FeMoS cluster complex catalyzes electrolytic production of pyruvic acid from an acetyl thioester and CO_2_ in non-aqueous solution. A thioester is required because other acetylated compounds do not yield pyruvic acid. Lactic acid is not reported to be a product [114]. Pyruvic acid is also produced under simulated hydrothermal vent conditions from alkyl thiols and CO in the presence of transition metal sulfides at 250 °C [37]. Hence, TPP-independent synthesis of pyruvic acid from an acetyl-thioester and CO_2_ catalyzed by FeS is possible.

Carbonyl groups of aldehydes and ketones react with thiols to form thioacetals and thioketals and the reaction does not proceed further to form hydroxyl groups and disulfides. Indeed, this reaction is employed for protection of aldehydes and ketones in organic syntheses [115]. In contrast, imino groups add thiols and the reaction can proceed to produce amino groups and disulfides [116,117]. Carbonyl groups in glyoxylate and pyruvate could have been converted into imines with NH_4_^+^ and then into Gly and Ala with thiols, regenerating disulfides. Synthesis of amino acids by reductive amination of α-ketoacids occurs in the presence of FeS and NH_4_^+^ or amines, but not in the presence of Fe(II) or other transition metal sulfides [118], and Ala is produced in mixtures containing pyruvic acid/FeS/H_2_S/H_2_/NH_4_Cl [108].

Glycine synthesis could also have involved reactions similar to those catalyzed by glycine synthase [119] in which NH_4_^+^, formaldehyde, and CO_2_ are converted into Gly with oxidation of dihydrolipoamide to the disulfide form. Formation of aminomethyllipoamide is non-enzymatic [119], pyruvic acid may have been involved instead of pyridoxal pyrophosphate (PLP) [120], and reductive carboxylation of imines to amino acids has been demonstrated electrolytically [121,122]. Non-enzymatic synthesis of Gly by transamination of glyoxylic acid using Ala in the absence of PLP is also known [123].

Among the earliest four-carbon molecules synthesized in primitive cells would have been oxaloacetic, malic and aspartic acids. Malic acid is synthesized in biology by condensation of acetyl-CoA with glyoxylic acid catalyzed by malate synthase [124], reductive carboxylation of pyruvic acid by malic enzyme [125,126], hydration of fumaric acid by fumarase [90], and reduction of oxaloacetic acid by malate dehydrogenase [127]. A non-enzymatic malate synthase-like route to malic acid would require base catalyzed formation of the enolate anion of an acetyl-thioester. Another path to malate may have involved intermediate formation of oxaloacetate, which is synthesized biochemically using pyruvate carboxylase, a biotin dependent carboxylase using bicarbonate [128]. In this reaction, the enolate anion of pyruvic acid is stabilized prior to interaction with a carboxyl group. The enol form of oxaloacetate is stabilized by an FeS center [129]. Non-enzymatic synthesis of malic acid from oxaloacetic acid, perhaps by reduction of FeS-bound oxaloacetic acid, would require formation of a C–H bond in the same way as reduction of pyruvic acid to lactic acid. FeS/H_2_S reduces oxaloacetic and pyruvic acids to malate and lactate [109]. Reductive amination of oxaloacetic acid would yield Asp.

Simple carboxylic acids containing thiol groups could have been available in primitive cells. Reductive thiolation of a Schiff base formed between an amino acid and glyoxylic acid could have yielded thioglycolic acid. This method is used to introduce thiol groups into coenzymes M and B [130,131]. Thiolactic acid is one of the products of the reaction of pyruvic acid and FeS/H_2_/H_2_S/CO_2_ [108].

The feasibility of reducing carboxylic acids to aldehydes for aldol condensation reactions is not well established in FeS cluster chemistry, but a reduced aldol condensation product is obtained from pyruvic acid/FeS/H_2_/H_2_S mixtures [108]. This result implies an ability of the FeS surface to stabilize the enol or enolate form of pyruvic acid. Stabilizing enols may be a property of FeS clusters. Fumarase A catalyzes the isomerization of enol and keto isomers of oxaloacetic acid [129].

In conclusion, the available evidence suggests that reduction and other reactions that are catalyzed by FeS or involve oxidation of thiols to disulfides could have led to the synthesis of molecules inside a primitive cell, including Gly and Ala for α-polypeptide synthesis, and malic acid for β-polyester synthesis. Activation of amino and hydroxy carboxylic acids as thioesters would facilitate acyl group transfer and polymerization.

## 15. Base Catalysis in Early Metabolism

Some of the earliest anabolic reactions would have involved synthesis of three- and four-carbon molecules. It is possible that early reaction mechanisms differed considerably from enzyme reaction mechanisms. For example, greater use may have been made of CO instead of CO_2_ in carbon fixation. If modern mechanisms do reflect the earliest mechanisms, base catalyzed removal of protons would have been advantageous for many reactions. Acid/base catalysis is one of the most important functions provided by enzymes. The imidazole side chain of His, and the carboxylic acid side chains of Asp and Glu are the most widely used acid/base catalytic groups in proteins. An extractable fraction of tholin contains terminal carboxyl and aminoacyl groups [67], and some of these groups would be expected to locate on the inside of the tholin membrane facing the FeS particle. Hence, tholin might also have supplied an early acid/base catalytic function through –COOH/COO–. For example, bringing a thioester and a catalytic base into proximity would be important for Claisen condensation reactions involving intermediate formation of enolates of acetyl-thioesters. Malate synthase uses this mechanism with glyoxylic acid as substrate and Asp as the catalytic base for formation of the enolate anion of acetyl-CoA [124].

## 16. A Model for the Simple System within a Primitive Cell

**Figure 2:**
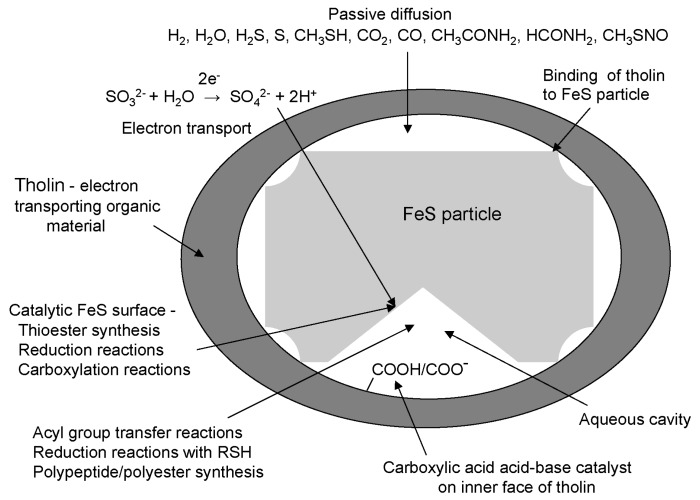
Model of a primitive cell. An FeS particle is enclosed in an organic membrane composed of tholin. Electron transport across the membrane to the FeS particle catalyzes thioester formation, carboxylation, and reduction reactions at the particle surface. Disulfides are reduced to thiols during thioester synthesis and regenerated through reduction reactions for further thioester synthesis. Transfer of acyl groups from thiol to hydroxyl and amino groups drives formation of polyesters and polypeptides. Carboxylic acid groups on the inside surface of tholin provide acid/base catalysis.

The preceding analysis supports the proposal that FeS may have acted as a mediator of reduction reactions and thioester synthesis as proposed by Wächtershäuser [21], but suggests that these reactions occurred using FeS as a catalyst within an organic membrane formed from tholin. Electrons released by the oxidation of sulfite could have been transported through tholin and delivered to FeS bound to tholin through thiol-groups. Freshly prepared particles of FeS have a highly rugged surface and contain a “library of different FeS clusters” [132]. Cavities between the FeS particle and tholin could have contained water. A model is proposed for the reactions within such a primitive cell that led to the synthesis of polypeptides and polyesters (Figure 2). This would be a simple system from which biology could evolve. FeS would be a true catalyst because it would not be converted into FeS_2_. The flux of reducing power into these cells allowed thioesters rather than ATP to be the main source of chemical energy for polyester and polypeptide synthesis and thiols rather than NADPH and reduced ferredoxin to be the mobile source of reducing power for synthesis in primitive metabolism. The interior of primitive cells would be reducing, as it is in all extant cells.

## 17. Genetic Polymers Were Initially Produced from Aliphatic Monomers

So far, I have not mentioned genetic polymers. Previous experimental work on the evolution of nucleic acids has focused primarily on simplifying the nucleic acid backbone, leaving the purines and pyrimidines for base pairing (reviewed in [133]). At the hub of metabolism are the carboxylic acid cycles, suggesting that the metabolites of early cells would have been carboxylic acids and their amino, hydroxy, keto, and thioester derivatives. All of these molecules are aliphatic. Aromatic molecules and molecules with aromatic components have long biosynthetic pathways starting from aliphatic molecules. Therefore, purines and pyrimidines, the aromatic amino acids, the quinone components of ubiquinone and menaquinone, nicotinamide of NADH, isoalloxazine of FADH_2_, folic acid, pterin ring systems, and other aromatic compounds are unlikely to have been involved in cellular activity until pathways for their syntheses had evolved.

**Figure 3:**
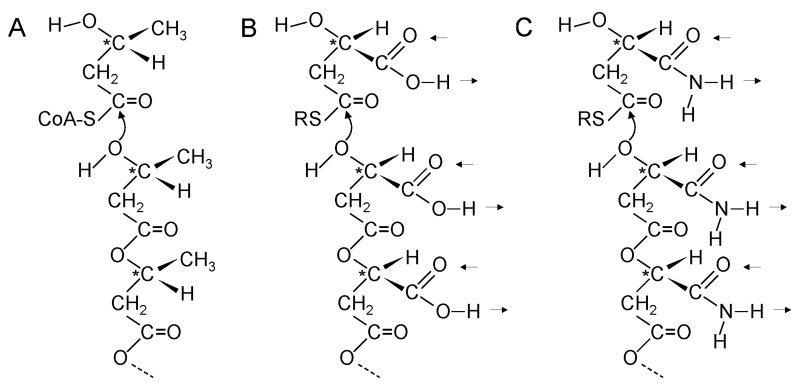
β-Polyester synthesis. (**A**) Enzyme catalyzed synthesis of poly(β-l-hydroxybutyric acid). The CH_3_ side chains in poly(β-l-hydroxybutyric acid) occur at repeating distances along the polyester chain and point in the same direction relative to the backbone; (**B**,**C**) Proposed non-enzymatic synthesis of poly(β-d-malic acid) (B) and poly(β-d-malamide) (C) in primitive cells. The double hydrogen bonds of the side chains occur at repeating distances and are in the same direction relative to the backbone. Arrows indicate directions of possible hydrogen bonds. * indicates chiral carbon atom.

Hydrogen bonding between the purines and pyrimidines of nucleic acids is the foundation of genetics. How did genetics start if purines and pyrimidines were not available from the environment or from synthesis inside early cells? Consideration must be given to the possibility that genetic polymers started with aliphatic side chains rather than purines and pyrimidines. Returning to the structure of PHBA, which is a simple linear β-polyester formed from β-l-hydroxbutyryl-CoA monomers, it is interesting that the methyl groups occur at repeating positions along the polyester chain and the homochiral nature of the polymer ensures that the methyl side chains all point in the same direction relative to the polyester backbone (Figure 3A).

Could a simple β-polyester with a different side chain have formed interstrand hydrogen bonds? The simplest functional groups in organic chemistry that can form at least two roughly parallel hydrogen bonds are carboxylic acids and carboxamides. Malic acid is a β-hydroxyacid and the simplest β-polyester with carboxylic acid side chains is water soluble poly(β-malic acid) [134,135,136] (Figure 3B). Most notably, malic acid is a central component of the carboxylic acid cycles. Consequently, it is likely to be been available early in the origin of life. β-polyesters formed from a single optical isomer are more hydrolytically stable than those formed from a racemic mixture and are more stable than α-polyesters [137]. Hence, chiral polymers would accumulate preferentially. Homochiral poly-β-malic acid would have carboxylic acid side chains all pointing in the same direction relative to the backbone. If d-malic acid were used for β-polyester synthesis it would be the predecessor of d-ribose that is found in the backbone of RNA. l-malic acid is used primarily in the carboxylic acid cycles.

A carboxyl group can accept hydrogen bonds via its oxygen atoms and donate a hydrogen bond through its hydroxyl group. Two hydrogen bonds are used when a carboxylic acid forms an intermolecular hydrogen bonded cyclic dimer. Dimerization is possible when the carboxyl group is unionized, which might have been the case under the slightly acid conditions on the early Earth resulting from a high pressure of CO_2_ [138]. Does poly(β-d-malic acid) form cyclic double hydrogen bonds between the carboxylic acid side chains producing a double stranded polyester in water? At present, there is no direct evidence to answer this question. Studies of poly(β-l-malic acid), which presumably has properties similar to poly(β-d-malic acid), have not distinguished the types of hydrogen bonding [139]. Formation of interstrand hydrogen bonds between carboxyl groups would be expected to involve a balance between hydrogen bonding to another carboxyl group and hydrogen bonding to water, similar to interstrand hydrogen bonding in DNA. Double stranded DNA is converted into single stranded DNA by increasing the temperature above the melting temperature. Bases in single stranded DNA form hydrogen bonds with water. The melting temperature for a double-stranded poly(β-d-malic acid) would likely be considerably lower than that of double stranded DNA which has triple hydrogen bonded GC base pairs and has been optimized for base pairing during the evolution of DNA.

Some carboxylic acids, including malic acid [140], crystallize with cyclic double hydrogen bonds between carboxyl groups and others, including acetic acid [141], crystallize with a catemer motif in which each carboxyl hydrogen bonds to two others in a chain. However, in water acetic acid forms mainly cyclic dimers [142]. Lactic acid dissolved in water is also present as the cyclic dimer [143]. Indeed, the cyclic dimer seems to be the lowest energy state for a carboxylic acid in water Consequently, hydrogen bonding between carboxyl groups is preferred over hydrogen bonding to water at ambient temperatures. If the carboxylic acid side chains of poly(β-d-malic acid) behave in a similar manner, interstrand double hydrogen bonding would be preferred over single stranded hydrogen bonding with water. They might even form cyclic double bonds cooperatively. However, the enthalpy gained by hydrogen bonding would have to offset the loss of entropy involved in creating a more ordered double stranded structure. In summary, it is possible that poly(β-d-malic acid) forms interstrand double hydrogen bonds especially at low temperatures and under slightly acidic conditions.

**Figure 4:**
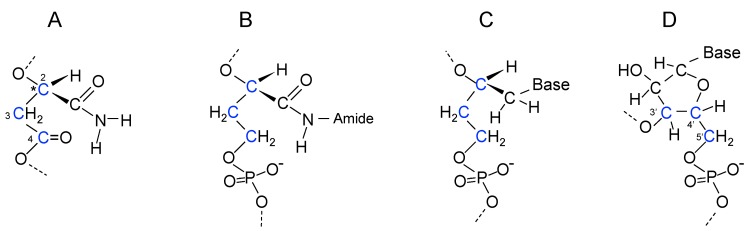
Evolutionary pathway proposed for the RNA backbone. (**A**) A malamide monomer in poly(β-d-malamide). C2, C3, and C4 carbon atoms are highlighted in blue; (**B**) Reduction of the 4-carboxyl group of malamide to a hydroxymethyl group forms d-2,4-dihydroxybutyramide allowing phosphodiester linking of monomers; (**C**) Reduction of the carboxamido carbonyl group of d-2,4-dihydroxybutyramide to a methylene group produces a d-3-deoxyerythritol backbone that forms phosphodiester links and binds to amines or heterocyclic bases through C1; (**D**) Replacement of the d-3-deoxyerythritol backbone by 3'-5' phosphodiester linked d-ribose. Conservation of the C2, C3, and C4 atoms of malamide as the 3', 4', and 5' carbon atoms of ribose is shown in blue. Evolution of the polymer side chains into pyrimidines and purines is discussed in Section 18 in relation to Figure 5A,B and Figure 6. * indicates chiral carbon atom.

The hydroxyl group of malic acid evolved into the 3'-OH of ribose and the β-carboxylic acid into the 5'-CH_2_OH. An early evolutionary development for the side chain may have involved use of malamide thioester monomers instead of malic acid thioester monomers (Figure 3C). Proposed steps in the evolution of the β-polyester backbone into the phosphoribose backbone of RNA are shown in Figure 4. Incorporation of phosphate into the backbone required evolution of a mechanism for phosphate uptake and utilization, possibly by reaction with an acetyl-thioester to generate acetyl-phosphate. The ribose-phosphate backbone was selected because it allowed the fully aromatic bases in RNA to stack at the distance and positions required for maximum stability.

## 18. Evolution of the Purines and Pyrimidines of Nucleic Acids from Aliphatic Side Chains

**Figure 5:**
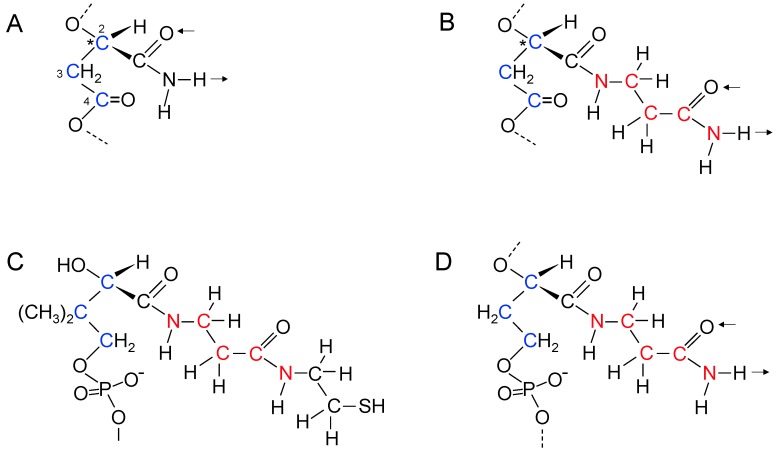
Poly(β-d-malamide) as an information polymer. (**A**) Malamide in a β-polyester strand with backbone carbon atoms shown in blue and amide side chain double hydrogen bonding shown by arrows. The 2', 3', and 4' carbon atoms of malamide are labeled and highlighted in blue; (**B**) Proposed extended side chain of malamide with β-alaninamide in a polyester strand. Carbon and nitrogen atoms in the extended side chain are highlighted in red. Hydrogen bonding is in the same direction relative to the backbone as that shown in A; (**C**) Structure of the phosphopantetheine component of CoA; (**D**) Structure of a proposed intermediate in nucleic acid evolution with a phosphodiester-linked backbone and extended malamide side chain, showing the similarities to phosphopantetheine. Two methyl groups in phosphopantetheine are not present in the proposed nucleic acid predecessor. * labels the chiral carbon atom.

An information polymer has to have at least two different side chains. Poly(β-d-malic acid) is not an information polymer. The carboxylic acid side chains of poly(β-d-malic acid) are obviously a long evolutionary distance from purines and pyrimidines and the pathway to them would involve multiple steps, which will be described in the following paragraphs. As mentioned previously, the first step could have been the use of d-malamide instead of d-malic acid, which would have provided a unionizable side chain (Figure 3C and Figure 4A). Transamidases catalyze conversion of carboxylic acids into amides with Gln often acting as the ammonia donor [144]. In the primitive setting, amides such as acetamide and formamide may have provided the amide NH_2_. In the absence of enzymes, transamidation reactions occur in the presence of Fe(III) and water using different NH_2_-containing reagents [145]. Modification of this amide side chain with a β-carboxamide such as Asn or β-alaninamide formed by α-decarboxylation of Asn could have produced a longer side chain that could form a double hydrogen bond in the same direction as the unmodified side chain (Figure 5A,B). At this point, the β-polyester formed from monomers with two different side chains became an information polymer. Hydrogen bonding between polyester strands using different amide side chains allowed “amide pairing”.

*De novo* synthesis of pyrimidines and purines presents a conundrum. Many of the steps are dependent upon energy provided by ATP or GTP hydrolysis. These enzymes may have replaced earlier enzymes that used a simpler energy source such as pyrophosphate, acetyl-phosphate, phosphoenol pyruvate, or a thioester. The reactions in purine synthesis that formylate the amino group of 5-aminoimidazole-4-carboxamide and then cyclize the product to form inosine with loss of water do not involve ATP hydrolysis and might have occurred non-enzymatically or with simple polypeptides as catalysts, perhaps using a formyl-thioester as substrate. Two enzymes in pyrimidine synthesis, carbamoyl-phosphate synthase and CTP synthase, use ATP as a substrate, and two other steps involve complicated enzymatic mechanisms. Overall, evidence from biochemistry does not provide many clues to explain the evolution of pyrimidine and purine synthesis. Nevertheless, I will propose pathways starting with the extended side chain shown in Figure 5B.

**Figure 6:**
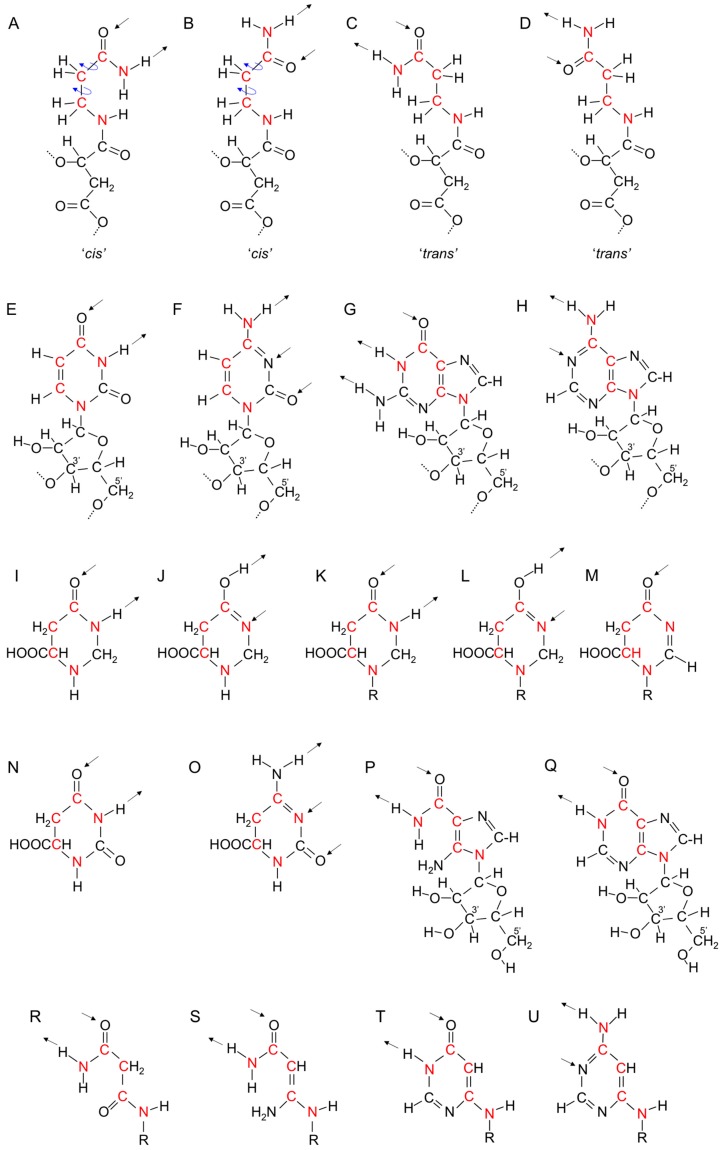
Evolution of purines and pyrimidines. Arrows show directions of possible hydrogen bonds. (**A**–**D**) Structures of the malamide side chain extended with β-alaninamide in “*cis*”- and “*trans*”-like conformations relative to the two CH_2_ groups. Blue arrows indicate rotations producing different conformations. Nitrogen and carbon atoms in the extended side chain are highlighted in red; (**E**–**H**) Structures of ribonucleic acid bases in RNA, (uracil, cytosine, guanine, and adenine) showing their relationships to the “*cis*”- and “*trans*”-like structures shown in A–D; (**I**,**J**) Epimers of tetrahydroorotate, a possible intermediate in pyrimidine evolution formed by reaction of Asn with formaldehyde; (**K**,**L**) Tetrahydroorotate epimers linked to the backbone of nucleic acid predecessors; (**M**) A pyrimidine derivative, formed by crosslinking of Asn using formylation, lacking the N–H bond at position 3 needed for double hydrogen bonding; (**N**) Dihydroorotate intermediate in uracil synthesis; (**O**) 4-Aminodihydroorotate, a proposed evolutionary predecessor of cytosine; (**P**) 4-carboxamido-5-aminoimidazole ribonucleotide intermediate in purine synthesis; (**Q**) Inosine, the first purine ring synthesized in the biochemical pathway to adenine and guanine; (**R**,**S**) Proposed intermediates in the evolution of the six-membered ring of purines lacking the imidazole ring. The carbonyl group of malonamide (R) is converted to an amino group (S) using the same reactions used in inosine biosynthesis; (**T**) Proposed predecessor of inosine. Six-membered pyrimidine ring is linked through a 6 amino group to the nucleic acid backbone. The amino and amido nitrogen atoms of S are linked via formylation; (**U**) Proposed predecessor of adenine. T is converted into an amino derivative similar to the conversion of inosine into adenine.

Examination of the structures of purines and pyrimidines suggests the way in which the modified aliphatic side chains evolved into purines and pyrimidines. In Figure 5 and Figure 6, carbon and nitrogen atoms in the extended aliphatic side chain are highlighted in red. Rotations allow the side chain to adopt conformations that are *cis*- and *trans*-like with respect to the two methylene groups of β-alaninamide and form double hydrogen bonds in two conformations (Figure 6A,B,C and D). The evolutionary pathways to the bases involve cyclization of the *cis*-like side chain to form pyrimidines and cyclization on each side of the *trans-*like side chain to form purines (Figure 6E,F,G and H).

The general approach to ring formation in pyrimidine and purine synthesis is to form carbon-nitrogen bonds by removal of water. Asn or β-alaninamide might be expected to provide the hydrogen bonding atoms at the 3 and 4 positions of pyrimidines. Indeed, one of the simplest ways of making a pyrimidine ring is the reaction of Asn with formaldehyde. Cyclization by crosslinking the amino and amido groups yields a tetrahydropyrimidine ring, tetrahydroorotate, which exists as two epimers in equilibrium [146,147] (Figure 6I,J). The amido group is trapped in the “*cis*” form in a heterocyclic, but not aromatic, intermediate in the evolution of pyrimidine synthesis. Cyclization of Asn or β-alaninamide side chains could produce tetrahydroorotate rings bound to the backbone of a nucleic acid predecessor (Figure 6K,L). The two epimers form double hydrogen bonds similar to the two amide conformations of the extended “*cis*” amide (Figure 6A,B). Hence, at this point cyclization did not distinguish between the two amide conformations. The strategy for ring closure employed in purine synthesis involves amino group formylation followed by ring closure with an amide NH_2_ and loss of water. When this strategy is applied to cyclization of Asn or β-alaninamide, N–H bonds at both the 1 and 3 positions of the ring are not produced and double hydrogen bonding is not possible for a backbone bound pyrimidine ring (Figure 6M).

Biochemical pyrimidine synthesis generating an aromatic ring starts with Asp rather than Asn. Use of carbamoyl phosphate, a rather unstable reagent produced using ATP, allows cyclization with loss of water to leave an amide-like N–H at position 3. The additional C=O in the ring makes a dihydrouracil derivative with a more unsaturated and nearly planar ring that would be expected to exist preferentially as one epimer (Figure 6N). This step would have needed uptake and use of phosphate, perhaps as acetyl phosphate [34]. Further reactions to produce the aromatic pyrimidine ring require complicated enzymatic reactions. Flavin mononucleotide is required for conversion of –CH_2_–CH(COOH)– to –CH=C(COOH)–, and a decarboxylation reaction involves stabilization of a carbene [148,149]. The last steps in the evolution of uracil synthesis may have occurred after steps were taken in purine synthesis.

A pyrimidine base that is specific for pairing with inosine or its predecessor required synthesis of an exocyclic amino group similar to that found in cytosine. Biochemically, this step follows formation of uracil. One possibility is that the unsaturated ring shown in Figure 6N was converted into a ring containing an exocyclic amino group (Figure 6O), before the complicated reactions evolved to produce the aromatic pyrimidine rings of uracil and cytosine. This dihydropyrimidine ring would pair with inosine or its predecessor.

Trapping of the “*trans*” amide form of β-alaninamide or Asn into a ring by crosslinking of the nitrogens with formaldehyde or formic acid is not possible. Purines are fusions of five and six-membered rings. The six-membered ring, which happens (confusingly) to be a pyrimidine ring, contains the hydrogen bonding function for base pairing. The “*trans*” carboxamide side chains (Figure 6C,D) are cyclized in the six-membered rings of purines (Figure 6G,H). However, purine biosynthesis assembles the five-membered imidazole ring first and builds the six-membered ring on the imidazole ring. The carboxamide group that provides the hydrogen bonding atoms of inosine is inserted into the imidazole ring. A carbonyl group in the imidazole ring, originating from Gly, plays a role in making the six-membered ring because prior to insertion of the carboxamide group it is converted into an amino group that is formylated for cyclization with the carboxamide NH_2_ to produce inosine (Figure 6P,Q).

The sequence of events in purine biosynthesis—imidazole ring first, pyrimidine ring second—may mirror the sequence of events in the evolution of purine synthesis. If this were the case, the synthetic steps in imidazole ring synthesis evolved prior to the introduction of the carboxamide group, presumably for a function other than purine synthesis. Another possibility is that the imidazole ring was incorporated into the reaction sequence for purine synthesis after introduction of the carboxamide group allowed the imidazole ring to be a substrate for the reactions leading to the six-membered ring. Therefore, an alternative, shorter evolutionary pathway for synthesis of the six-membered ring could have preceded assimilation with the imidazole ring. Crosslinking of the trans-amide conformation into a six-membered ring would require similar introduction of an amino group in the side chain corresponding to the amino group of the imidazole ring. A malonamide side chain in place of Asn or β-alaninamide could have supplied the carbonyl group that was converted into an amino group for cyclization (Figure 6R,S). This evolutionary development would have required the ability to synthesize malonic acid, perhaps in the absence of biotin, by carboxylation of the enol form of an acetyl-thioester.

A substituted pyrimidine ring with double hydrogen bonding capability linked to the backbone via a six-amino group could then be formed via formylation, which introduces unsaturation into the ring and makes it aromatic (Figure 6T). The formylation method could not be used for cyclization of a predecessor of uracil because protonation of N1 was required for linking of the ring to the backbone leaving N3 unprotonated and unable to be involved in hydrogen bonding (Figure 6M), but it can be used for cyclization of ring shown in Figure 6T because N1 is not used for linking to the backbone. The evolutionary predecessor of inosine shown in Figure 6T probably existed as one primary epimer and therefore double hydrogen bonding was similar to one of the two “*trans*” amide conformations, (compare Figure 6T with Figure 6C). It could have been a substrate for the reactions that convert inosine into adenine that could have introduced an exocylic amino group in place of oxygen (Figure 6U), allowing double hydrogen bonding similar to the other “*trans*” amide conformation (compare Figure 6U with Figure 6D). Oligomers containing 2,4-disubstituted pyrimidines linked through a five-amino rather than a 6-amino group base pair weaker than biological bases, leading to the observation that “canonical nucleobases represent a functional optimum with respect to informational base pairing in aqueous solution” [150]. Subsequent inclusion of the imidazole ring would have improved base pairing. Cytosine and its proposed predecessor had the capacity to form a triple hydrogen bond, and guanine synthesis evolved from inosine to provide a triple hydrogen bonding partner, thereby increasing the strength of double stranded RNA interactions.

## 19. Coenzyme A as a Relic of Early Nucleic Acid Evolution

Synthesis of CoA, one of the most ancient molecules in biology, uses a pyruvoyl-dependent α-decarboxylation reaction for synthesis of its β-alanine component from Asp [151]. The phosphopantetheine component of CoA is shown in Figure 5C. Remarkably, its structure has similarities to the extended side chain and an intermediate in RNA backbone evolution in which the 4-carboxylic acid of malic acid has been reduced to a hydroxymethyl group that allows formation of phosphodiester bonds (Figure 4B and Figure 5D). The main difference is the presence of the two CH_3_ groups in the phophopantetheine. Perhaps, these groups prevented pantetheine from being recognized by the ancient proteins involved in activation of monomers for polymerization. The structure of CoA seems to contain a relic of the early evolution of nucleic acids.

## 20. Coupling of β-Polyester Synthesis and Protein Synthesis

The mechanism for synthesis of PHBA suggests that activation of malic acid and malamide monomers for polymerization could initially have involved formation of β-thioesters. Polymerization would proceed with the terminal OH group attacking the thioester bond of the next monomer (Figure 3B,C). Because the OH group is the predecessor of the 3'-OH of ribose, the direction of synthesis is equivalent to the 5' to 3' direction in RNA polymerization. Aminoacyl thioesters form polypeptides in the absence of enzymes [27,28]. Could these two processes be combined to allow coupled synthesis of proteins and polyesters? As noted earlier, acyl groups move from thiol to hydroxyl groups [26] that would allow formation of aminoacyl esters from aminoacylthioesters. Unlike a simple ester bond such as that in ethylacetate, aminoacyl ester bonds have energies similar to thioester or phosphoanhydride bonds [152], and it is the energy in aminoacyl-tRNAs that drives peptide bond formation in ribosomal protein synthesis [153,154]. Consequently, aminoacyl group transfer from an aminoacyl thioester to the OH of malate or malamide thioesters would create aminoacylated monomers that could also be used for polymerization of β-polyesters. Aminoacylation of the OH group of malic acid or malamide would be the evolutionary predecessor of aminoacylation of the 3'(2')-OH of ribose by the aminoacyl-tRNA synthetases.

At a time when the coding system of ribosomal protein synthesis was beginning to be revealed, Rich [155] proposed that life started with coupling of nucleic acid and amino acid polymerization using a singlet coding system that could have initially involved only two nucleotides, each of which was ester linked to a specific amino acid. In the context of polymerization of β-polyesters and proteins, this idea can be adopted for coupling of ester bond formation for extension of the β-polyester chain with peptide bond formation for extension of the protein chain. If two different monomers, with extended (=M’) and unextended (=M) side chains preferentially aminoacylated with Gly and Ala, respectively, a simple singlet coding system would be created for coupled synthesis.

HOOC-M’-M-M’-M_-Ala-Gly-Ala-Gly_ + M_-Ala_ → HOOC-M’-M-M’-M-M_-Ala-Ala-Gly-Ala-Gly_

**Figure 7:**
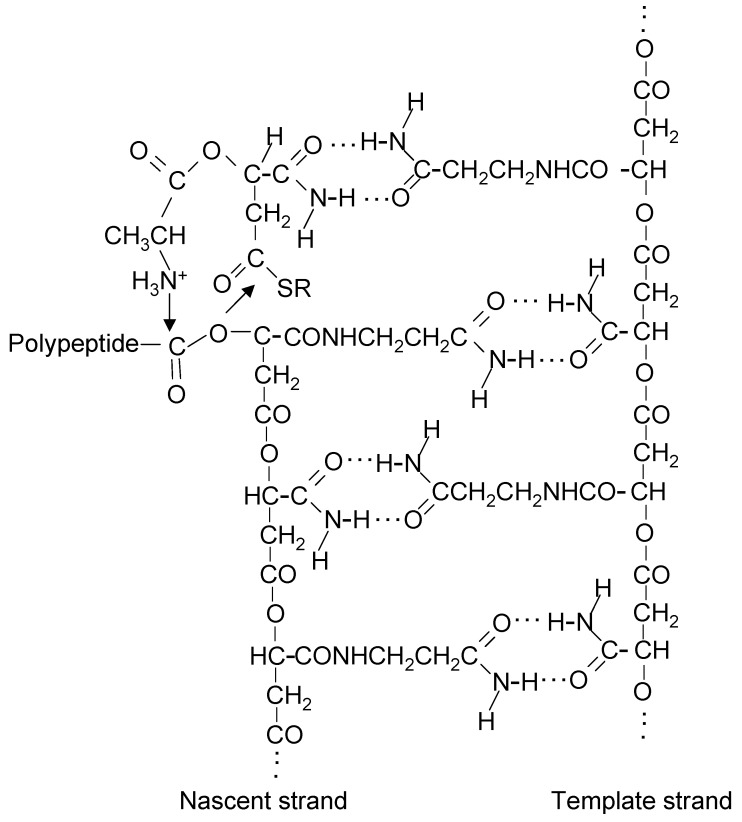
Coupled synthesis of poly(β-malamide) and polypeptide. Extension of a nascent poly(β-malamide) strand using a poly(β-malamide) strand as template and an alanyl-malamide thioester monomer. The monomer amide pairs with an extended malamide side chain on the template strand. Formation of the ester bond is accompanied by transfer of the polypeptide at the end of the nascent strand to alanine producing a new peptide bond. The leaving group is a thiolate anion. Not shown are loss of a proton from the alanyl NH_3_^+^ group and gain of a proton by the thiolate anion to produce a thiol.

This would have been the first mechanism for coded protein synthesis. Peptide bond synthesis in this process would resemble that of diglycine directed by polyU from 2'(3')-glycyl esters of adenosine derivatives [156]. Figure 7 shows addition of an alanyl-malamide thioester monomer to a nascent β-polyester strand using another polyester strand as template for amide pairing. The polypeptide at the end of the nascent strand is transferred to alanine when the new ester bond is formed.

Although insertion of proteins into membranes appears to be an unlikely primitive process, insertion of segments synthesized from a small number of simple amino acids such as Gly and Ala would have been a simpler method for membrane biogenesis than synthesizing lipids. Gly-Ala polypeptides inserted into tholin were the first membrane components to be synthesized. Another, far less studied, membrane component, PHBA, forms pores in membranes [157] and also has a shorter synthetic pathway than lipids.

## 21. A Thiol at the N-Terminal End of Early Proteins

In the preceding analysis, the thiol group occupies central functions in formation of thioesters and disulfides, and as a ligand to FeS compounds. The thiol group is also a strong nucleophile. Hence, it may have been advantageous, and perhaps essential, to have a thiol group in the earliest protein sequences. However, only the simplest two amino acids, Gly and Ala, were coded in the first singlet system. Cys appears to have been a late addition to the triplet coding system [158,159]. Eck and Dayhoff [85] in a study of ferredoxin evolution suggested that a primitive sequence containing Ala, Asp, Ser, and Gly evolved by duplication. Later, Cys was added to the sequence allowing binding to FeS and then as more Cys were added binding to Fe_4_S_4_. “On the principle that evolution proceeds one step at a time we assume that the cell was already using iron sulfide as a catalyst probably attached to cysteine alone or to some peptide less suitable than protoferredoxin”. What was this peptide? The question can be restated: How could a thiol have been incorporated into the singlet coding system? Met/fMet is the initiating amino acid in protein synthesis. Why has such an unusual amino acid assumed such a prominent function? Met is synthesized from homocysteine. Therefore, homocysteine may have been the initiating amino acid in the triplet coding system prior to incorporation of Cys into proteins, and may also have been the evolutionary successor to a thiol at the N-terminal end of proteins in the early singlet process. By being at the N-terminal end the simple initiating thiol did not necessarily have to be an amino acid. A simple acid such as thioglycolic acid or thiolactic acid may have been sufficient. A thiol at the N-terminal end of all proteins allowed them to bind to the FeS particle and be involved in catalyzing transfer of acyl groups and reduction reactions at the FeS surface. Later, assembly of FeS clusters enabled cells to become independent of FeS particles while retaining critical FeS-mediated functions.

The catalytic function of carboxylates of tholin was taken over by the C-terminal ends of the polypeptides inserted into the membrane material. Early Gly-Ala polypeptides could have brought together a thiol group at the N-terminal end and a carboxylic acid at the C-terminal end for acid/base catalysis. Gly-rich loops supplied an anion binding function [159].

## 22. Autocatalysis Revisited

As mentioned previously, autocatalysis with symmetry breaking in biology depends upon the use of proteins composed of l-amino acids to catalyze production of proteins composed of l-amino acids. It is now possible to propose how symmetry breaking in biochemistry began. Activation of malamide monomers by aminoacylation with simple amino acids such as Gly and l-Ala was catalyzed by simple proteins made from Gly and l-Ala. The amino acid binding site would preferentially bind l-Ala over d-Ala. When this activation process evolved to allow preferential binding of l-Ala to the malamide monomer with the unextended side chain and binding of Gly to the malamide monomer with the extended side chain, a simple coding system became possible that allowed conservation of amino acid sequence and configuration, and symmetry breaking was achieved.

## 23. Replication of β-Polyesters

Because different malamide side chains would be capable of forming double hydrogen bonds, a simple replication process would involve using one strand of β-polyester to act as a template for synthesis of a complementary strand (Figure 7). Duplicated sequences, like gene duplications, would allow selection for improved or changed protein functions. However, in a singlet coding system mutations in base sequence lead directly to changes in amino acid sequence. There are no silent mutations. Multiple copies of genetic sequences would be needed to evolve new protein sequences but also to mitigate the deleterious effects of mutations that altered amino acid sequences.

## 24. Evolution of the Singlet Coding System into the Triplet Coding System

As the polyester backbone evolved into the phosphoribose backbone of RNA and the purine and pyrimidine bases evolved, the singlet coding system allowed four bases to code for four amino acids (Gly, Ala, Asp, Val). The triplet coding system of ribosomal protein synthesis evolved from the singlet system permitting RNA synthesis and protein synthesis to be uncoupled and multiple copies of protein to be synthesized using a single strand of RNA as template. If the four codons to be used in ribosomal protein synthesis were GNC (N = any base), the second base would determine the amino acid (U = Val, C = Ala, A = Asp, G = Gly) allowing continuity with the singlet coding system [5]. Evolution of the standard genetic code from an early code that included only these four amino acids is widely supported [5,8,159,160,161,162,163].

## 25. Overview of the Evolution of Nucleic Acid Synthesis, and the Origin and Evolution of the Genetic Code

Synthesis of the four bases in RNA did not evolve at the same time. As far as the aromatic bases are concerned, uracil biosynthesis precedes cytosine and inosine precedes both adenine and guanine. The first products are those in which the “amide NH_2_”, not the “amide C=O”, is incorporated into the rings. Evolution of base pairing must take this into account. Moreover, some steps in biosynthesis of the bases appear to be more difficult than others. Uracil synthesis has some difficult steps, as do the syntheses of cytosine from uracil and guanine from inosine. This hypothesis adopts the position that the four bases were not obtained from the environment but were the products of evolutionary processes within cells. Prior to synthesis of the aromatic bases, intermediates in their syntheses were used in pairing. These intermediates would also not have become available at the same time.

By combining the analysis in this study with those given previously [5,153], an overview of nucleic acid base evolution, amide/base pairing, and the origin and evolution of the genetic code can be presented, starting with the singlet coding system and ending with the triplet coding system:

(1) carboxamide side chain (Figure 5A), coding for Ala, pairs with extended carboxamide side chain (Figure 5B), coding for Gly.
(2) tetrahydropyrimidine derivative (Figure 6K or Figure 6L), coding for Ala, pairs with extended carboxamide side chain (Figure 5B), coding for Gly,
(3) tetrahydropyrimidine derivative (Figure 6L), coding for Ala, pairs with inosine predecessor (Figure 6T), coding for Gly,
(4) tetrahydropyrimidine (Figure 6L), coding for Ala, pairs with inosine predecessor (Figure 6T); tetrahydropyrimidine (Figure 6K), coding for Ala, pairs with adenine predecessor (Figure 6U), coding for Asp.
(5) uracil predecessor (Figure 6N) coding for Val, pairs with adenine predecessor (Figure 6U), coding for Asp; cytosine predecessor (Figure 6O), coding for Ala, pairs with inosine predecessor (Figure 6T), coding for Gly,
(6) uracil (Figure 6E), coding for Val, pairs with adenine (Figure 6H), coding for Asp; cytosine (Figure 6F), coding for Ala, pairs with inosine, coding for Gly (Figure 6Q)
(7) uracil (Figure 6E), coding for Val, pairs with adenine (Figure 6H), coding for Asp; cytosine (Figure 6F), coding for Ala, and pairs with guanine (Figure 6G), coding for Gly.
(8) Codons: GUC codes for Val, GCC codes for Ala, GAC codes for Asp, GGC codes for Gly. The second base in the codon, underlined, is the same as the base in the singlet coding system.
(9) Expansion of genetic code to include 20 amino acids.

A biochemical example of a carboxamide double hydrogen bonding with uracil is found in the structure of photolase where uracil binds to the carboxamide of Asn (Figure 4 in [164]).

## 26. Discussion

### 26.1. An Evolutionary Pathway from Primitive Cells to Biology

The events that enabled a simple cellular system to assemble on the primitive Earth and evolve into the complex system of biology are difficult to understand. The four major activities of modern cells—membrane synthesis, energy transduction, catalysis, and replication of genetic polymers—have to be considered together. The “metabolism first” approach to the origin of life leaves out the genetic component and the “genetics first” approach leaves out the metabolism that was needed to support synthesis of genetic polymers. In this paper and others in this series [5,8,159], I have used chemistry and biochemistry to propose an evolutionary pathway from the first cells, using molecules found at the center of metabolism for synthesis of simple linear polyesters and polypeptides, to the complex metabolism of biology with nucleic acid synthesis and ribosomal protein synthesis.

### 26.2. Comparisons with Other Proposals on the Origins of Cells

The origin of cells described here and earlier [8] bears similarities and differences with other proposals for primitive cells. It is similar to proposals that the first cells had membranes composed of organic molecules [165] but, in agreement with Folsome [66], uses tholin rather than single chain lipids as the organic material. The proposal resembles the “lipid world” hypothesis [166] in that the membrane material, tholin, played an important early function by binding to FeS through thiol groups and supplying carboxylic acid groups for acid/base catalysis. It is similar to proposals that FeS surfaces provided reactive sites for reductive synthesis [21,167] but differs in that FeS (a) was not converted into FeS_2_; (b) was enclosed in thiol-containing tholin that facilitated electron transport and diffusion of small non-polar and polar molecules while only allowing slow diffusion of ions; and (c) catalyzed formation of thioesters and other molecules using energy from oxidation reactions such as oxidation sulfite to sulphate. The idea of encapsulating a mineral, including FeS, in an organic enclosure has precedents in other studies [168,169,170].

### 26.3. Why Is the Simple Method of Thioester Synthesis Not Used in Biology?

If FeS-mediated thioester synthesis occurred by a simple mechanism such as that shown in Figure 1, why is there no evolutionary footprint of this mechanism in modern cells? The proposed mechanism is non-specific. While such a mechanism may have been useful initially it may have become superseded by protein-catalyzed reactions that provided higher levels of specificity. An FeS catalyzed reaction may have supplied a mechanism for thioester synthesis but a side reaction reducing efficiency may have been reduction of a disulfide to thiols similar to an electrolytic reduction of a disulfide [171]. This level of efficiency may have been inadequate as evolution proceeded. The model presented involves a simple thiol such as CH_3_SH and other simple thiols, thioglycolic or thiolactic acids at the N-terminal end of proteins. The different functions of thiols in redox reactions, metal binding, and carrying acyl groups and NO, have largely become specialized in the course of evolution. This may also have contributed to the loss of the primitive mechanism for thioester synthesis.

### 26.4. Coded Proteins Enabled Evolution of Nucleic Acid and Ribosomal Protein Synthesis

A widely held view is that prior to evolution of ribosomal protein synthesis catalysis of chemical reactions was performed by RNA (reviewed in [133]). Catalysis by RNA is strongly dependent upon the properties of RNA including the presence of the 2'-OH group, triple hydrogen bonding by GC base pairing, and A-minor interactions. RNA is an extremely complex polymer. If simpler evolutionary predecessors of RNA did not have a ribose-phosphate backbone with its 2'-OH or GC base pairing, the catalytic properties of those polymers would have been different from those of RNA. Yet, it would have been these polymers that would have been responsible for catalyzing RNA synthesis [172]. A more plausible explanation is that coded protein synthesis was accomplished early in the origin of life beginning with Gly and Ala and then Gly, Ala, Val and Asp in a singlet coding system. As a result, proteins would have been available to catalyze reactions needed for evolution of RNA synthesis and ribosomal protein synthesis, and to facilitate energy transduction, membrane biosynthesis, and membrane transport.

### 26.5. The Need for Simplicity of Metabolism, Catalysis, and Replication in the Primitive Cell

In critiques of “metabolism first” ideas about the origin of life, Ross [173] and Orgel [174] note two particular difficulties with metabolic cycles as precursors to genetics. One is that chemical reactions generally have low specificity and increased side reactions compared to enzyme catalyzed reactions. Reactions of a particular type, conversion of a carboxylic acid into a carboxamide for example, might apply to all carboxylic acids. Lactate formation from pyruvate would be a side reaction that would impede carboxylic acid cycles in metabolism first scenarios. The other difficulty is that most syntheses under presumed prebiotic conditions yield a complex mixture of products. Side reactions reduce the efficiency of chemical reactions. Orgel [174] suggests that, “Simplification of product mixtures through the self-organization of organic reaction sequences, whether cyclic or not, would help enormously, as would the discovery of very simple replicating polymers” and “… a theory in which metal sulfide-catalyzed reactions provided some or all of the organic molecules needed for the formation of a primitive genetic system would have many attractive features”. In addition, it has been concluded that catalysts in the origin of life should allow accumulation of large concentrations of a few components rather than low concentrations of many components [175]. In the proposed primitive cells, the number of substrates is limited by restricting diffusion across tholin membranes of ions and large polar molecules, and the types of chemical reactions catalyzed by FeS is limited. The end products of electron flow into the cell are polyesters and polypeptides. Poly(β-d-malic acid) is a simple predecessor of the nucleic acids. One functional group, a thiol, acts in redox reactions, carries activated carboxylic acids, and binds to Fe. Therefore, the needs for a simple primitive metabolic system and a simple replicating polymer are met by this hypothesis. If nucleic acid synthesis began with malic acid and its amide derivatives, the “genetics first” and “metabolism first” approaches to the origin of life are reconciled because malic acid features prominently in carboxylic acid cycles. However, the reverse citric acid cycle was probably too complex to have been established at the beginning.

### 26.6. Reversible Reactions in Primitive Cells

Some enzyme-catalyzed reactions in cells operate under near equilibrium conditions and others are essentially irreversible. Interconversion of lactic acid and pyruvic acid catalyzed by lactate dehydrogenase is a near equilibrium reaction and can operate in each direction dependent upon the conditions, especially the concentrations of lactic and pyruvic acids. Was the ability of reactions to be reversible also a feature of primitive cells where the catalysts for reactions were FeS and the inner surface of the tholin membrane? The study by Wang *et al.* [109] shows that some reduction reactions may have been reversible. An FeS/H_2_S redox system reduces glyoxylic, pyruvic and oxaloacetic acids into the corresponding hydroxy acids, glycolic, lactic and malic acids. If this were irreversible, pyruvate would be “trapped” in lactic acid and could not be available for synthesis of other molecules including malic acid. But when sulfur is added to the mix with FeS and H_2_S, the hydroxy acids are oxidized to glyoxylic, pyruvic and oxaloactic acids, showing that the reactions are reversible. This would allow some synthetic pathways to be favored over others. In this proposal, the two most favored pathways are those leading to β-polyester and α-polypeptide synthesis.

### 26.7. The Four Bases in Nucleic Acids Are Derived from the Four Orientations of Carboxamide Side Chains

The four bases used for pairing in nucleic acids are not the only ones that could have been used [176]. Why use a genetic alphabet with just four? Szathmáry [177] suggests that the bases are a relic of the RNA world. The explanation that comes out of the proposed evolution of the pyrimidine and purine bases is that the four bases evolved from the two possible orientations of the carboxamide group in the *cis*- and *trans*-like carboxamide evolutionary intermediates (Figure 6A,B,C and D).

### 26.8. Did “Chemical Evolution” Happen before Biological Evolution?

There is a long history to the idea that biological evolution with a genetic selection or Darwinian capability was preceded by a period of “chemical evolution” [178,179]. In the course of chemical evolution, reactions occurred in aqueous solution (the prebiotic soup) or through wet-dry cycles, driven by the energy intrinsic to certain chemicals, such as cyanides and aldehydes, by photolysis, or by surface catalysis, to produce pathways leading to increasingly complex metabolic cycles, polypeptides and oligonucleotides. It is then assumed that these chemicals became encapsulated in lipid vesicles initiating biological evolution [180]. Leaky vesicles enabled diffusion of nutrients and replication [181]. The hypothesis presented here supports the view that there was not a period of chemical evolution. Life started with energized cellular structures and the molecules necessary for biological evolution were generated within these structures from simple molecules that diffused into the cells. Evolution began with the formation of the first cells.
